# Modeling the effect of drug courts in North Carolina counties

**DOI:** 10.21203/rs.3.rs-8704196/v1

**Published:** 2026-02-16

**Authors:** Nikolas Lindauer, David Kline, Samrachana Adhikari, Amanda M. Bunting, Staci A. Hepler

**Affiliations:** 1 Department of Statistical Sciences, Wake Forest University.; 2 Department of Biostatistics and Data Science, Wake Forest University School of Medicine; 3 Department of Population Health, NYU Grossman School of Medicine

**Keywords:** opioid overdose, courts, spatial, Bayesian, intervention, autoregressive

## Abstract

**Background.:**

Illicit opioid overdose death rates drastically increased across North Carolina through 2023. We sought to study the impact of drug courts and what conditions can make their presence more or less effective.

**Methods.:**

We analyzed counts of illicit opioid overdose deaths for each county in North Carolina from 2017 – 2023. Bayesian Poisson autoregressive models are used to model the change in illicit opioid overdose death rates. We included an indicator of drug court presence in the model and used interaction terms to quantify effect heterogeneity. We used our model to estimate counterfactual outcomes and quantify the effect of drug courts.

**Results.:**

We found drug courts were associated with decreases in illicit opioid overdose death rates, and that the effect was heterogeneous across North Carolina. We estimated a large protective effect in counties with fewer drug possession arrests, after controlling for other covariates.

**Conclusion.:**

Diversion into drug courts can provide an effective pathway to reducing the overdose crisis, but the effectiveness of drug courts depends on other features of the county.

## 1 Background

Between 1999 and 2023, over 800,000 people died from a drug overdose, with the number of deaths in 2023 being nearly ten times the number in 1999 (Centers for Disease Control and Prevention, 2025). Of the 105,000 overdose deaths nationwide in 2023, approximately 76% involved opioids. The rapid increase in opioid involved overdoses observed since the early 2010’s is largely attributed to illicit opioids including heroin and fentanyl. Many individuals with substance use histories also have contact with the criminal legal system (CLS). From 2015–2019, an estimated 37.2% of adults with past-year CLS involvement also had a substance use disorder (Shah et al., 2024). Throughout this period, the prevalence of drug arrest among people who use illicit drugs was relatively stable and estimated at approximately 2.0% in metro areas; however, estimated prevalence increased from approximately 3.0% in 2015 to over 5.0% in non-metro areas (Rouhani et al., 2024).

While illicit opioid use is prevalent both among those who have contact with CLS and also among those who experience drug overdose, evidence as to the relationship between arrests and overdoses is inconsistent throughout the literature. Between 2013 – 2017, increased opioid-related arrests were associated with decreases in opioid-involved overdose death rates in Michigan counties (Rydberg et al., 2019). On the contrary, an analysis of data from the Madison Addiction Recovery Initiative concluded the traditional policing approach to drug use-related crime was associated with a risk of future overdose fatalities (Zhang et al., 2022). Misdemeanor arrest rates in New York City precincts were associated with higher rates of overdose mortality between 1990–1999 after adjusting for overall drug use (Bohnert et al., 2011). In Marion county, Indiana, opioid-related law enforcement drug seizures were significantly associated with increased overdoses (Ray et al., 2023). Possible causal mechanisms to explain the positive associations between drug-related law enforcement activity and overdose rates have been hypothesized. Fear of police has been cited as a reason why people who use drugs frequent areas which are harder for both police and medical services to access and as a reason someone observing an overdose might not call emergency services, both of which can result in increased overdose deaths (Tracy et al., 2005; Bohnert et al., 2011). Reduced tolerance following a period of imprisonment has been identified as a cause of increased risk of overdose death following CLS involvement (Binswanger et al., 2013).

Many lawmakers have shifted from a criminal model of using arrests as a means of reducing opioid use, instead prioritizing the treatment of individuals who use drugs paired with the criminalization of those who traffick or manufacture drugs (Sacco, 2018). One intervention is the institution of drug courts, which first appeared in the United States in the 1980s and now can be found in all 50 states (Csete, 2020). Drug courts serve as an alternative to criminal courts in which those found in possession of small amounts of illegal drugs, if referred by their local elected prosecutor, may pursue treatment instead of face incarceration. Drug courts meet routinely to ensure the patient is adequately adhering to their treatment goals, typically involving drug tests, frequent counseling, and potentially additional requirements such as ending associations with other persons with CLS involvement, maintaining employment, or treatment via methadone or buprenorphine (National Association of Drug Court Professionals, 2024).

Numerous studies have analyzed the success of drug courts, focusing primarily on the individuals who participate in the programs and their outcomes. Although nearly half of drug court participants do not graduate from the program (Randall-Kosich et al., 2022), rates of recidivism are drastically reduced among participants (Ekstrand et al., 2005; Mihaly, 2016; Mitchell et al., 2012). Cost and benefit data from a small number of drug treatment programs found that the reduction in recidivism saves local justice systems between $1,000 and $15,000 per drug court participant (Ekstrand et al., 2005). Despite this, variation in funding, eligibility criteria, policies, and procedures undermine the effectiveness of many drug courts (Easter et al., 2021; Ekstrand et al., 2005). While there is substantial evidence that drug courts reduce recidivism, the relationship between drug courts and overdose has not been well-studied. One exception is Lindenfeld et al. (2022), who conducted a difference-in-difference study using national county-level data from 2000 – 2012 to conclude that drug courts significantly reduced overdose mortality. However, the time frame of that study predates the rapid increase in opioid-related overdose which coincided with the rise in fentanyl (Spencer et al., 2024).

The aim of this study is to quantify the relationship between drug courts and illicit opioid overdose death rates in North Carolina counties. We used data from 2017 – 2023 and state-of-the-art Poisson autoregressive models to model the yearly change in illicit opioid overdose death rates as a function of drug courts and other covariates. We hypothesized that the effect of drug courts is heterogeneous and related to other demographic, socioeconomic, and political features of the county, and thus we fit a second model to better understand this effect heterogeneity.

## 2 Methods

### 2.1 North Carolina Data

The outcome variable for our analyses was county-level illicit opioid overdose deaths for each year from 2017–2023, obtained from the North Carolina Division of Public Health’s Overdose Epidemic Data dashboard (NC Department of Health and Human Services Division of Public Health Injury & Violence Prevention Branch, 2025). These counts are determined by ICD-10 codes X40-X44 and T-codes T40.1 or T40.4, summarizing unintentional illicit opioid poisoning deaths (World Health Organization, 2004). During the period studied, most opioid overdose deaths were due to heroin, fentanyl, or some analogue of the two, but overdose deaths due to hydrocodone or other commonly prescribed opioids were excluded from this count. Deaths were recorded based on the county of the victim’s residence, not the location of the overdose itself.

**Figure 1:**
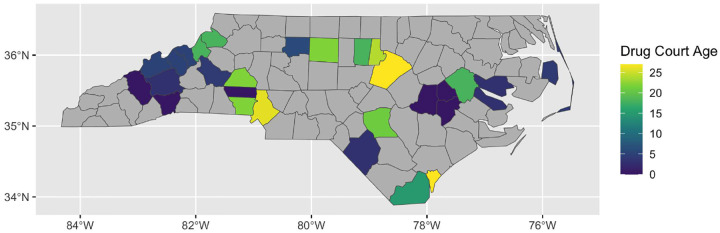
Length of time that a county has had a drug court as of 2023. Note that the gray counties do not have a drug court.

The exposure of interest was whether or not each county had a drug court in that year. Figure 1 shows how long each county’s drug court had been in operation as of 2023. Note that newer courts are along the western border and coast while the older courts tend to be located in major population centers such as Mecklenburg (Charlotte) and Wake (Raleigh) counties. The counties in gray are those without a drug court. We can see that most counties in NC did not have a drug court as of 2023.

We extracted data from a variety of publicly available sources with the intent to study heterogeneity in the effect of drug courts. We considered socioeconomic variables, variables which measure access to healthcare, variables that reflect police activity within the county, among others. Table 1 lists all variables utilized and their sources. Each variable is available for 2018–2023 for each county.

Motivated by literature on the complex relationship between arrests and overdoses, we constructed a variable that quantified the extent to which each county’s law enforcement was focused on possession offenses. We developed a metric dubbed the targeting index, which we defined as the number of drug possession arrests (excluding marijuana) divided by the total number of arrests for crimes against persons or crimes against property for each county for each year (North Carolina State Bureau of Investigation, 2025). Dividing by total arrests adjusts for variation in overall police activity. We discretized the targeting index into three bins with cutoffs based on the tertiles so that the low targeting bin refers to counties in the bottom third (targeting index below 0.107) and high targeting for counties in the highest third (targeting index above 0.217). Maps of the targeting bins for each county can be found in Supplemental Figure 1. We additionally included an indicator for High Intensity Drug Trafficking Areas (HIDTA) designation. This designation identifies areas where drug-related activities have a significant harmful impact (U.S. Drug Enforcement Administration, 2025).

### 2.2 Statistical Methods

We reduced the number of highly correlated explanatory variables by constructing two indices – a poverty index and a healthcare accessibility index. The poverty index is the first component of a principal components analysis using the percent of each county who are in poverty, unemployed, disabled, receive food stamps and did not complete high school. The weights that specify the linear combination of these variables is in Supplemental Table 1 and indicate that higher values of the poverty index correspond to worse socioeconomic conditions. The healthcare accessibility index is the first component of a principal components analysis using the percent of each county that are uninsured, an indicator if the county is a medically underserved area, and the number of licensed opioid treatment providers in each county. The weights for each variable are in Supplemental Table 2 and indicate higher values of the index correspond to worse access to healthcare.

We used a Poisson autoregressive model (Fokianos and Tjøstheim, 2011) to quantify the relationship between drugs courts and the *change in* expected overdose deaths per capita each year. The autoregressive structure allowed each county to have its own baseline expected death count, enabling estimation of how covariates relate to the change in the rate, relative to the initial magnitude for that particular county. After the initial year, all subsequent years’ average death rates were modeled with the previous year’s rate as an offset, so that the exponentiated regression coefficients quantify the death rate ratios between consecutive years. We assumed county-specific intercepts modeled with an intrinsic conditional autoregressive spatial random effect to account for spatial autocorrelation and to allow each county to have its own average rate of change.

We considered two different models. The first model sought to estimate the effect of a drug court. This model included the socioeconomic covariates listed in Table 2 as main effects as well as an indicator of whether the county had a drug court in that year. Since we suspected the relationship between drug courts and change in overdose deaths may differ by other features of the county, we considered a second model that included interaction effects between the drug court indicator and all covariates to quantify heterogeneous effects. Both models were fit in a Bayesian framework. The full model description can be found in Supplemental Material Section 2.

To contextualize and interpret the effect and potential that drug courts have for reducing illicit opioid overdose deaths, we simulated counterfactual outcomes under three hypothetical scenarios, and we compared the counterfactual illicit death counts and rates to what was actually observed. Changes in the death counts were calculated by taking the differences between counterfactual and observed death counts for each year, and then summing over the years. Changes in death rates were obtained by dividing the change in counts by the county’s population from that year, then averaging over each year. We summarized the statewide impact by estimating the total change in death counts and average change in death rates for the state, where the total change in death counts is the sum of the county-level counts described above, and the average change in rate is the average of the aforementioned county-specific change in rates, averaged across all 100 NC counties.

The counterfactuals were computed as the estimated mean of the posterior predictive distribution under each scenario using posterior samples of the parameters from the model described above. First, we considered the scenario where we assume North Carolina had no drug courts during this time period. This allowed us to estimate the effect that the current drug courts have had on illicit opioid overdose deaths. Our second counterfactual assumed that all counties had a drug court for the duration of the study, allowing us to estimate the impact that universal adoption of drug courts would have. Finally, in line with our theory that high targeting is detrimental to the success of drug courts, we constructed one more counterfactual in which each county has both a drug court and all high targeting indicators are removed, and we compared the predicted death counts and rates under this scenario to what was actually observed.

## 3 Results

**Figure 2:**
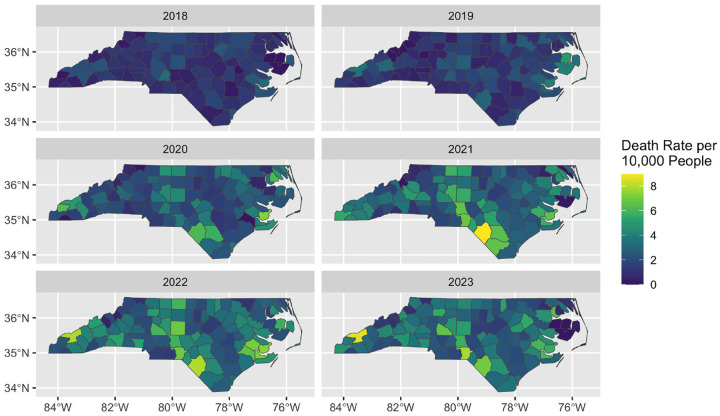
Observed illicit opioid overdose death rates per 10,000 residents by county and year

Figure 2 contains the observed illicit opioid overdose death rate for each county for 2018 – 2023. Death rates increased dramatically across the state, especially along the southern border and the western half of the state into Appalachia. Summary statistics for each variable, summarized over counties with and without drug courts, as well as the standardized mean differences, are available in Supplemental Figure 3.

Our first model estimated a 95% probability that the presence of a drug court is associated with greater decreases in illicit opioid overdose deaths when controlling for our covariates. Specifically, we estimated a rate ratio of 0.97 in year-to-year changes relative to an identical county without a drug court, or an annual 3% reduction. There is over a 95% posterior probability that the rate ratio is less than 1.0. A table of all covariate estimates and credible intervals for model one can be found in the supplementary material.

Our second model that included interaction effects found the effect of a drug court was heterogeneous and modified by covariates. Drug courts for counties where all other variables are zero were associated with an average yearly decrease of 16% relative to expected values for these counties without drug courts. This relationship was weakened significantly in the presence of high targeting of drug possession offenses, which our model predicted to remove any beneficial association between drug courts and death rates. On the contrary, low values of the healthcare index were associated with more productive drug courts, indicating a potentially protective effect that drug courts have in areas with poor healthcare access that we would otherwise predict to be doing worse. Table 2 contains the estimated rate ratios and credible intervals for the drug court indicator and all interaction effects, thus quantifying how the overall effect of drug courts varied. Estimates of the regression coefficients for the non-interaction covariates can be found in the supplementary material.

**Figure 3:**
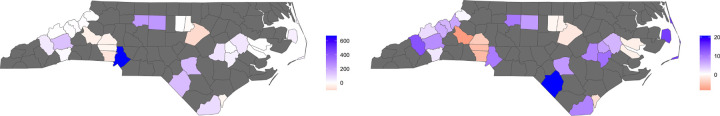
These maps show the estimated impact of drug courts compared to the first counterfactual that assumed no drug courts. (Left) Estimated change in overdose death counts over the study period; (Right) Estimated average change in deaths per 10,000 population. Note that counties which did not have drug courts are gray.

Figure 3 shows the estimated change in total overdose deaths and change in average rate for each county under the first counterfactual where we compare to the scenario of no drug courts. Note that positive values correspond to an estimated increase in deaths if there are no drug courts, as compared to what was actually observed. Unsurprisingly, we generally estimated that counties with drug courts would have experienced more overdose deaths had there not been drug courts. However, there were several counties with negligible or even slightly negative estimates. These counties were generally high income with good healthcare access and few actual overdose deaths, and thus have lower likelihood to benefit relative to the observed values.

**Figure 4:**
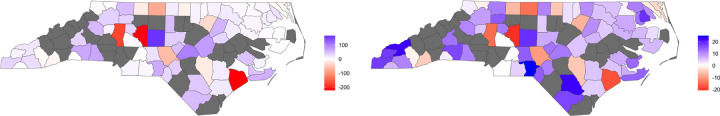
Estimated impact of the establishment of drug courts in each county, aggregated over the study period. The left map represents the reduction in overdose death counts. The right map represents the average change in deaths per 10,000 population. Note that counties which already have drug courts are gray.

Next, we compared the observed death counts to counterfactual counts assuming universal adoption of drug courts. Figure 4 contains maps of the estimated change in overall deaths and average rate aggregated over the 5 year span. Positive values indicate the presence of a drug court was associated with reduced deaths. Most counties were estimated to have a reduction in illicit opioid overdose deaths had drug courts been established, but there are a few counties where the estimated effect is negative. These counties tended to have high targeting and/or very low observed opioid overdose rates.

**Figure 5:**
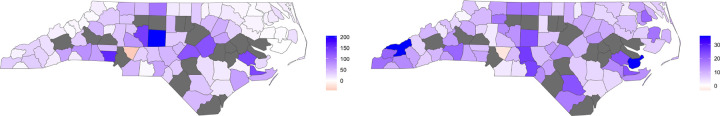
Estimated impact of the establishment of drug courts with no high targeting in each county, aggregated over the study period. The left map shows the estimated decrease in overdose death counts while the right map represents the estimated decrease in deaths per 10,000 population. Note that counties which already have drug courts and are not high targeting are gray.

Lastly, we compared the observed death counts to predicted values under the counterfactual scenario that every county had a drug court but did not have high targeting. Figure 5 contains maps of the estimated change in overall deaths and average rate. Under this scenario, we estimate widespread benefits. The only county which did not have estimated benefit was Cabarrus county, which instead showed a slight increase in overdose deaths. Notably, Cabarrus, as an urban county with high socio-economic status and high healthcare access, was the only NC county whose illicit opioid overdose death rate generally decreased during the observation window.

Overall, the estimated statewide impact over the five year window is summarized in Table 3. The table provides the estimated difference in death counts and average death rate under the three counterfactual scenarios (ordered such that positive values indicate a protective effect of drug courts), as well as 95% credible intervals. Our model predicted that existing NC drug courts are associated with a reduction of 1641 illicit opioid overdose deaths between 2018 and 2023, with the average statewide death rate reduced by 1.226 deaths per 10,000 individuals. If the remaining counties had similarly enacted drug courts, we estimated an additional 21 deaths would have been prevented, and an average reduction in death rate of 4.259 per 10,000. We also estimated that the cessation of high targeting practices by police and the establishment of a drug court in each county would be associated with a decrease of an additional 2821 deaths over the observation window, with a change in reduced average death rate of 9.719 per 10,000.

## 4 Discussion

It is well-established that drug courts reduce recidivism among participants. Existing literature has found that the effects on recidivism and drug use vary substantially across jurisdictions (see, e.g. Wilson et al. (2006); Rossman et al. (2011)). This variation has been attributed to programmatic differences including eligibility criteria, funding, and prosecutorial discretion, underscoring the importance of local context in shaping outcomes (Koozmin, 2016; Randall-Kosich et al., 2022). However, the effects of drug courts on opioid overdose mortality are not well-studied. Lindenfeld et al. (2022) found that drug courts significantly reduce county-level drug overdose mortality, but they did not analyze effect heterogeneity. In this study, we used a Poisson autoregressive model with multiple interaction terms to understand the extent to which county-level features are associated with more or less successful drug courts in North Carolina. We defined success as a reduction in the yearly multiplicative change of illicit opioid overdose deaths.

Our findings extend the literature by demonstrating that the effectiveness of drug courts in reducing opioid overdose deaths is heterogeneous and strongly moderated by socioenvironmental and political factors. Specifically, drug courts were most effective in counties with poor access to healthcare. This is unsurprising, as areas with worse access to healthcare coincide with the areas historically most impacted by the opioid epidemic, and many drug court participants have a history of healthcare neglect (Garcia and Lucas, 2021). Easter et al. (2021) noted that in 2020 in North Carolina, which pre-dates the state’s Medicaid expansion, adult drug court participants were unlikely to be insured. Additionally, Best Practice Standards state drug courts should allow the use of all FDA-approved medication for opioid use disorder (National Association of Drug Court Professionals, 2015), and drug courts applying for federal funding through the Center for Substance Abuse Treatment cannot deny participants medication for opioid use disorder (Nordstrom and Marlowe, 2016; Marlowe et al., 2022). As such, in areas where healthcare access is limited, drug courts may serve as one of the few pathways to evidence-based treatment and can partially compensate for gaps in the healthcare system, amplifying their protective effect.

On the contrary, drug courts have no estimated protective effect in counties classified as ‘High Targeting,’ meaning the ratio of drug related arrests to arrests for crimes against persons or property was in the top third of all counties under all years of study. It is plausible that minimal enforcement of drug possession crimes might limit the ability of drug courts to treat people who use opioids (PWUO), but also that a maximalist approach might strain relations between the CLS and PWUO. Low targeting presented a weaker harmful association with overdose rates in the absence of drug courts, but this impact was erased in the presence of courts. High targeting, on the other hand, had a stronger harmful association with overdose deaths in counties without drug courts and a profoundly amplified relationship in counties with drug courts. The tension between PWUO and law enforcement in these counties may be nullifying the positive potential drug courts typically have for treatment. This finding is consistent with prior research linking punitive enforcement to elevated overdose risk (Bohnert et al., 2011; Tracy et al., 2005; Zhang et al., 2022). Fear of arrest and strained relationships with law enforcement may deter individuals from seeking help or calling emergency services during overdoses. Thus, while drug courts may aim to reduce harm, their success depends on broader CJS practices that either support or conflict with treatment-oriented approaches.

Our results also suggest weaker drug court effects in urban counties, counties with a Democratic DA, and those with high crime rates, though these associations were not statistically significant. Though our HIDTA indicator did not have a statistically significant interaction coefficient, the sum of the coefficients from the interaction and non-interaction terms were significantly negative (see Supplementary Material). This finding indicates that HIDTA counties with drug courts have significantly reduced multiplicative change in death rates compared to non-HIDTA counties without drug courts. Interestingly, increased poverty did not significantly modify drug court effectiveness. While this is in contrast to some prior findings (e.g. Randall-Kosich et al. (2022)), this may reflect the dominating influence of other factors such as healthcare access and policing strategies.

Our inability to conduct a randomized study forces us to speculate on the causal nature of drug courts using observational data which relies on a number of causal assumptions, such as exchangeability and the stable unit treatment value assumption. Although we thoughtfully included covariates that we believed to play a role, there may be other unmeasured confounders that relate to either opioid overdose deaths or the existence of a drug court that were unaccounted for. Another limitation of our analysis is a result of the inconsistency across drug courts. We considered a county to be treated provided a drug court existed in that county, but individual drug courts are likely highly variable in their implementation, and we are unable to account for this inconsistency using the available data. While estimating community-level effects using aggregate data is of public health interest, care must be taken to avoid the ecological fallacy.

## 5 Conclusion

This paper used various data sources to study the relationships between policing, drug courts, and illicit opioid overdose death rates in North Carolina counties. We used Poisson autoregressive modeling and found drug courts are associated with reducing illicit opioid overdose deaths in counties with specific legal infrastructure, primarily without high targeting and with HIDTA resources. Additionally, the presence of drug courts reverses trends in several predictors for increased opioid overdose covariates, including low targeting and poor healthcare access.

Taken together, these findings reinforce that drug courts are not a one-size-fits-all solution. Their success hinges on local conditions, including healthcare infrastructure, law enforcement practices, and programmatic features. Future research should explore mechanisms underlying these interactions, particularly the role of treatment availability and CLS relationships in shaping outcomes. Policymakers should consider tailoring drug court implementation to jurisdictions where enabling conditions, such as low targeting for drug-related crimes and reduced access to healthcare, maximize potential benefits. Conversely, in high-targeting environments, reforming policing practices may be necessary to unlock the harm-reduction potential of drug courts.

## Supplementary Material

Supplementary Files

This is a list of supplementary files associated with this preprint. Click to download.

- CodeFilesForSubmission.zip
- SupplementalMaterial.pdf

## Figures and Tables

**Table 1:** Each variable considered for our model is listed above, along with its source. NCAOC: North Carolina Administrative Office of the Courts; NCSBI: North Carolina State Bureau of Investigation; ONDCP: Office of National Drug Control Policy; ACS: American Community Survey; NCBE: North Carolina Board of Elections; DOA: Department of Agriculture; HRSA: Health Resources and Services Administration; SAMHSA: Substance Abuse and Mental Health Services Administration; NCDPH: North Carolina Division of Public Health

Variables	Sources
Drug Court Presence	NCAOC
Ratio of Possession Arrests to All Arrests	NCSBI
Arrests per Capita	NCSBI
High Intensity Drug Trafficking Area	ONDCP
Population	ACS
Percent of Households on Public Assistance	ACS
Percent of Households on Food Stamps	ACS
Percent of Households under Poverty Line	ACS
Percent of Potential Employees Unemployed	ACS
Percent of People under 65 Uninsured	ACS
Percent of People Disabled	ACS
Percent of People without a High School Diploma	ACS
District Attorney Political Affiliation	NCBE
Urban/Rural Designation	DOA
Medically Undeserved Area Designation	HRSA
Number of Opioid Treatment Providers	SAMHSA
Illicit Opioid Overdose Death Counts	NCDPH

**Table 2:** Estimated rate ratios for the drug court and drug court interaction terms, along with the lower and upper bounds of the 95% credible intervals. The last column is the estimated percentage of the posterior distribution that was greater than one. The variables that are bold correspond to regression coefficients where the 95% credible interval does not contain zero.

	Variable	Estimate	95% CI	Percent Positive
	**Drug Court**	0.84	0.77, 0.92	0.00
Interaction Effects	Low Targeting	0.96	0.90, 1.04	0.18
	**High Targeting**	1.21	1.09, 1.34	1.00
	Standardized Crime Rate	1.02	0.99, 1.05	0.90
	HIDTA	0.97	0.91, 1.02	0.13
	Democrat DA	1.05	0.99, 1.13	0.96
	Urban County	1.04	0.96, 1.14	0.83
	Poverty Index	1.00	0.98, 1.02	0.52
	**Healthcare Index**	0.95	0.92, 0.99	0.01

**Table 3:** Estimated state-level differences in death counts and death rates per 10,000 under each of the three counterfactual scenarios considered. 95% credible intervals are shown in parentheses.

Counterfactual Scenario	Change in Death Counts	Change in Rate
Impact of Current Courts	1641 (-39, 3316)	1.226 (0.2032, 2.260)
Universal Adoption of Courts	21 (-1251, 1329)	4.259 (1.266, 7.128)
Adoption and no High Targeting	2821 (1817, 3821)	9.719 (6.968, 12.22)

## Data Availability

The datasets supporting the conclusions of this article are included in the article’s supplementary files.
